# A retrospective case-cohort study comparing treatment outcomes in abacavir versus stavudine containing first line antiretroviral treatment regimens in children <3yrs old, at a paediatric programme based in Soweto, South Africa

**DOI:** 10.1371/journal.pone.0180645

**Published:** 2017-07-07

**Authors:** Haseena Cassim, Kennedy Otwombe, Erica Lazarus, Afaaf Liberty, Glenda E. Gray, Oppel B. W. Greeff, Avy Violari

**Affiliations:** 1 Perinatal HIV Research Unit, Faculty of Health Sciences, University of the Witwatersrand, Johannesburg, South Africa; 2 South African Medical Research Council, Parow Valley, Cape Town, Western Cape, South Africa; 3 Department of Pharmacology, University of Pretoria, Pretoria, South Africa; Katholieke Universiteit Leuven Rega Institute for Medical Research, BELGIUM

## Abstract

**Introduction:**

The current World Health Organization guideline for first line antiretroviral therapy (ART) in HIV-infected children recommends the use of abacavir and lamivudine as nucleoside backbones and no longer includes stavudine. We compared treatment outcomes with abacavir (ABC) versus stavudine (d4T) in a cohort of HIV-1 infected children 6 and 12 months after antiretroviral therapy was initiated.

**Methods:**

This was a retrospective case-cohort study, using programmatic data from children enrolled in the Paediatric Wellness Programme at the Perinatal HIV Research Unit in Soweto, South Africa between 2005 and 2013. Children on abacavir/stavudine who had initiated ART at age <3 years with a regimen including lamivudine and lopinavir/ritonavir and had at least one 6 or 12 month viral load result were eligible. All ABC cases identified were matched for age at ART initiation and gender to eligible d4T controls (1:2). Outcomes analysed at 6 and 12 months post ART initiation included virological failure, mortality, immunological failure and anthropometry. Chi-square tests compared categorical measures while Kruskal-Wallis compared continuous measures.

**Results:**

We identified 57 eligible ABC cases and selected 114 matched d4T controls. Overall, 57% were females and 89% started treatment at age <1year. The median age at ART initiation was 3.11 (IQR: 1.98–6.05) months. There was no difference in the proportion of children virologically suppressed between the groups at 6 (ABC 54.5% vs. d4T 67.0%, p = 0.125) and 12 (ABC 66.7% vs. d4T 71.6%, p = 0.53) months post ART-initiation. The proportion of children with adherence levels >90% for ABC and d4T were similar too (95% in ABC vs. 86% in d4T, p = 0.10). The proportion of children who died over 12 months was 3.5% in the ABC and 7.9% in the d4T group (p = 0.27). Similarly, the anthropometric measures were comparable.

**Conclusions:**

It is reassuring that in the short term, in this group of patients, the treatment outcomes were similar.

## Introduction

The management of paediatric HIV is accompanied by an abundance of challenges ranging from making the diagnosis, to the unwillingness of inexperienced clinicians in facing the challenge of paediatric HIV, to finding a suitable palatable formulation for children. It is not surprising then, according to the 2013 World Health Organization (WHO) progress report, that even though 630 000 children received treatment in 2012 compared to 566 000 in 2011, the percentage increase in children younger than 15 years receiving antiretroviral treatment (ART) was considerably less than the increase achieved amongst adults (11 vs 21%) [1]. Despite improvements in the prevention of mother to child transmission of HIV (PMTCT), there were still an estimated 2.6 million children under 15 years of age living with HIV in 2014 of whom only about 31% (800 000) were receiving antiretroviral therapy (ART) [2]. This paediatric treatment gap has been noted by the Joint United Nations Programme on HIV/AIDS (UNAIDS) and with improvements in early infant diagnosis, access to ART, and paediatric drug formulations, the global plan aims to increase paediatric ART coverage with the 90-90-90 treatment goal (90% of people living with HIV to know their HIV status, 90% of people who know their status to access HIV treatment and 90% of people on HIV treatment to achieve viral suppression) by 2020 [3].

ART reduces infant mortality by 75% when started before 12 weeks of age [4, 5]. In addition, the effectiveness of ART is indisputable in perinatally infected children surviving into adulthood. HIV has been transformed into a chronic disease, and as such, short and long term toxicities need to be considered when determining the best regimen. Earlier guidelines from the WHO for treating paediatric HIV, recommended using a nucleoside reverse transcriptase inhibitor (NRTI) backbone including a thymidine analogue—stavudine (d4T) or zidovudine (AZT) together with lamivudine (3TC) for children under 3 years of age [6]. Subsequently, the guidelines were changed to recommend substitution of the thymidine analogue with abacavir (ABC) based on studies which showed improved virological responses and fewer side effects with ABC compared to AZT or d4T [7–9].

In South Africa, the initial regimen for the treatment of HIV in paediatrics included the use of d4T as a first line NRTI based on earlier guidelines. In 2010, the updated WHO recommendations for ABC based therapy were implemented. However, there has subsequently been concern about the relative efficacy of ABC compared to d4T in Southern African children with conflicting results in the literature. Programmatic data from South Africa, both single site and multi-cohort, has indicated that early virological outcomes in ABC based regimens may be poorer than d4T based first line regimens [10, 11]. However, a randomised control trial (RCT), in African children, done in Uganda and Zambia found that AZT, d4T and ABC combined with 3TC and a Non-Nucleoside Reverse Transcriptase Inhibitor(NNRTI) all performed similarly with regards to clinical, immunological and virological outcomes [12]. While the RCT done in Uganda and Zambia used an NNRTI based regimen only, the reports on South African children included children on an NNRTI or a PI based regimen. In this study, we compared the treatment outcomes of ABC versus d4T in combination with 3TC and lopinavir/ritonavir (LPV/r) in a cohort of HIV-1 infected South African children <3years of age 6 and 12 months after ART initiation.

## Methods

### Setting

The Paediatric Wellness Programme (PWP) at the Perinatal HIV Research Unit (PHRU) in Soweto, South Africa is a prospective cohort of HIV-1 infected children, the majority of whom are perinatally infected. Since its inception in May 2005, the PWP has enrolled 2259 children, including patients who were diagnosed at infant and child testing facilities on site and in the community, as well as post-trial follow-up of children who completed participation in PHRU ART clinical trials. Up until November 2010, the NRTI backbone was d4T with 3TC and thereafter it was changed to ABC with 3TC.

Children are seen at least every 3 months. At each visit, anthropometric measures (weight and height); WHO staging and HIV and drug related events are assessed, and ART is given based on South African guidelines. CD4 count and viral load testing are performed at month 6 and month 12 post ART initiation, and annually thereafter.

### Study design and study population

This was a retrospective case-cohort study utilizing a subset of PWP participants. Children were eligible for inclusion if they were ART naïve when enrolled into PWP, initiated on ART at age <3 years with a regimen including 3TC and lopinavir/ritonavir (LPV/r) and had at least one 6 or 12 months post-ART initiation viral load. Subjects were excluded if they were participating or had previously participated in a clinical trial.

#### Selection of study population

A database search was done for all children on the PWP that had initiated an ABC containing regimen and were < 3 years of age. Thereafter, children were included if the background regimen was 3TC/LPV/r and had at least 1 viral load result at 6 or 12 months after treatment initiation (a window period of 3 months before and after the visit date was allowed). Children who had initiated a d4T containing regimen were similarly selected from the same database and matched to those in the ABC group by age and gender. As we had a bigger pool of d4T children, matching was done in a 1:2 (ABC: d4T) ratio. The age categories used for matching were 0–1, 1–2 and 2–3 years. The cases were matched randomly by computer programming. All the controls matched to a case were censored in order to avoid duplication in the matching process.

For the group receiving d4T, the visits ranged from 25 Aug 2005 to 6 Sep 2011 whereas the children in the ABC group were seen between 5 Nov 2010 and 28 May 2013.

The syrup formulation was used for all the drugs during the study period. Drug adherence was determined by a pharmacist by measuring the amount of drug returned and using this to calculate the quantity consumed compared to the quantity expected to have been consumed since last dispensing.

### Outcomes

Outcomes were virological failure (VF) defined as viral load ≥400 copies/ml, mortality, immunological failure defined as CD4 percentage ≤25%, and anthropometry: weight-for-age (WAZ), height-for-age (HAZ), weight-for-length (WLZ) z-scores. These were evaluated at baseline (pre-ART visit closest to ART initiation), 6 and 12 months post ART-initiation.

Adherence was considered as adequate if ≥90% of doses were taken.

### Ethical considerations

The PWP was approved by the University of Witwatersrand Human Research Ethics Committee (ethics reference number: M050455). Parents or caregivers of the participants provided written informed consent for participation and data collection. This retrospective analysis was approved by the University of Pretoria, Faculty of Health Sciences Research Ethics Committee (ethics reference number: 474/2015).

### Statistical analysis

Frequencies were determined for categorical variables whereas medians and interquartile ranges were determined for continuous measures overall and by treatment group (ABC or d4T). Continuous measures for anthropometric data such as height-for-age, weight-for-age, weight-for-length and BMI z-scores were categorised according to the WHO classification: < -2 or > -2. Categorical measures were compared by treatment group using chi-square analysis or Fishers exact test as appropriate whereas continuous measures were compared by the Kruskal-Wallis test. Virological, immunological and anthropometric outcomes were determined and similarly compared at 6 and 12 months. Additionally, anthropometric measures from baseline up to 12 months as well as their change from baseline were plotted. We also performed a sub-group analysis focusing on children with viral loads above 100,000 copies/ml.

The outcome virological failure was assessed at 6 and 12 months. The association between virological failure and treatment group controlling for baseline measures was assessed by the generalized estimating equations (GEE). In the GEE modeling framework, we controlled for matching, WAZ, participant age at treatment initiation and the year of treatment initiation (as a proxy for treatment trends). Additionally, we performed further analysis where virological failure was modelled using time varying covariates. Statistical analysis was conducted using SAS Enterprise Guide 7.1 (SAS Institute, Cary, NC)

## Results

We identified 57 eligible ABC cases and selected 114 matched d4T controls for a total sample of 171 children (56.7% female; 89.5% under 1 year of age at time of ART initiation) with a median age of 3.11 (IQR: 1.98–6.05) months (Table 1). Pre-ART, more children in the ABC group had viral load < 100,000 copies/ml (17.9% vs. 2.8%, p = 0.0013) and were catergorised as WHO stage 1 or 2 (86% vs 63.1%, p = 0.002) compared with the d4T group. However, the CD4% was similar between the groups. Children in the ABC group also had better median WAZ (-1.15 vs. -1.88, p = 0.0009) and WLZ (0.28 vs. -0.50, p = 0.0002) relative to d4T.

**Table 1:** Baseline characteristics.

Variables	Overall	ABC	d4T	P-Value
**Number enrolled**	171	57	114	
**Gender**				
Male (%)	74 (43.3)	27 (47.4)	47 (41.2)	0.44
Female (%)	97 (56.7)	30 (52.6)	67 (58.8)	
**Age (years) at ART start**				
0–1 (%)	153 (89.5)	51 (89.5)	102 (89.5)	-
1–2 (%)	15 (8.8)	5 (8.8)	10 (8.8)	
2–3 (%)	3 (1.8)	1 (1.8)	2 (1.8)	
**Median (IQR) age in months**	3.11 (1.98,6.05)	2.55 (1.95,4.86)	3.36 (2.05,6.25)	0.33
**Viral Load (copies/ml)**				
< 100,000 (%)	13 (7.9)	10 (17.9)	3 (2.8)	0.0013
≥ 100,000 (%)	152 (92.1)	46 (82.1)	106 (97.2)	
Log viral load	5.88 (5.58,5.88)	5.77 (5.28,6.35)	5.88 (5.76,5.88)	0.69
**CD4%**				
≤ 25% (%)	86 (50.3)	27 (47.4)	59 (51.8)	0.59
> 25% (%)	85 (49.7)	30 (52.6)	55 (48.2)	
Median (IQR)	25.00 (17.50,34.60)	25.32 (16.49,35.70)	24.55 (17.60,34.20)	0.799
**WHO Classification**				
Stage 1 or 2 (%)	119 (70.8)	49 (86.0)	70 (63.1)	0.002
Stage 3 or 4 (%)	49 (29.2)	8 (14.0)	41 (36.9)	
**Weight-for-Age z-score**				
< -2 (%)	69 (40.4)	15 (26.3)	54 (47.4)	0.008
≥ -2 (%)	102 (59.6)	42 (73.7)	60 (52.6)	
Median (IQR)	-1.66 (-2.80,-0.59)	-1.15 (-2.08,-0.29)	-1.88 (-3.00,-0.78)	0.0009
**Height-for-Age z-score**				
< -2 (%)	85 (49.7)	22 (38.6)	63 (55.3)	0.0399
≥ -2 (%)	86 (50.3)	35 (61.4)	51 (44.7)	
Median (IQR)	-1.93 (-2.90,-0.92)	-1.43 (-2.78,-0.78)	-2.20 (-3.28,-1.11)	0.0542
**Weight-for-Length z-score**				
< -2 (%)	23 (13.5)	4 (7.0)	19 (16.8)	0.0779
≥ -2 (%)	147 (86.5)	53 (93.0)	94 (83.2)	
Median (IQR)	-0.24 (-1.24,0.56)	0.28 (-0.45,1.09)	-0.50 (-1.42,0.21)	0.0002

The proportion of children virologically suppressed was similar between the groups at month 6 (ABC 54.5% vs. d4T 67%, p = 0.13) and 12 (ABC 66.7% vs. d4T 71.6%, p = 0.53) (Table 2). The median CD4% increased during follow-up in both groups but was not significantly different between them at 6 (ABC 30.68% vs. d4T 31.00%, p = 0.72) and 12 months (ABC 32.98% vs. d4T 31.90%, p = 0.62).

**Table 2:** Outcomes at 6 and 12 months.

Variables	6 Months			12 Months		
	ABC	d4T	P-Value	ABC	d4T	P-Value
**Viral Load**	55	100		51	102	
< 400 (%)	30 (54.5)	67 (67.0)	0.13	34 (66.7)	73 (71.6)	0.53
≥ 400 (%)	25 (45.5)	33 (33.0)		17 (33.3)	29 (28.4)	
Median Log_10_ VL (IQR)	2.44 (1.60,3.58)	2.60 (1.93,2.90)	0.28	2.24 (1.60,3.45)	2.60 (1.70,2.75)	0.20
**CD4%**	53	101		51	101	
≤ 25% (%)	14 (26.4)	27 (26.7)	0.97	11 (21.6)	16 (15.8)	0.38
> 25% (%)	39 (73.6)	74 (73.3)		40 (78.4)	85 (84.2)	
Median (IQR)	30.68 (24.76,38.20)	31.00 (24.80,35.70)	0.72	32.98 (26.39,38.90)	31.90 (28.00,37.40)	0.62
**Weight-for-age z-score**	56	103		56	104	
< -2 (%)	8 (14.3)	21 (20.4)	0.34	8 (14.3)	10 (9.6)	0.37
≥ -2 (%)	48 (85.7)	82 (79.6)		48 (85.7)	94 (90.4)	
Median (IQR)	-0.93 (-1.42,0.03)	-1.18 (-1.95,0.03)	0.18	-0.70 (-1.25,0.17)	-0.64 (-1.44,0.22)	0.93
Median change from baseline (IQR)	0.16 (-0.46,1.31)	0.76 (0.00,1.66)	0.004	0.26 (-0.37,1.32)	1.19 (0.40,2.09)	<.0001
**Height-for-age z-score**	56	103		56	104	
< -2 (%)	20 (35.7)	39 (37.9)	0.79	26 (46.4)	39 (37.5)	0.27
≥ -2 (%)	36 (64.3)	64 (62.1)		30 (53.6)	65 (62.5)	
Median (IQR)	-1.65 (-2.41,-0.67)	-1.58 (-2.65,-0.74)	0.67	-1.91 (-2.64,-1.23)	-1.72 (-2.41,-0.73)	0.25
Median change from baseline (IQR)	0.12 (-0.48,0.57)	0.34 (-0.37,1.14)	0.045	-0.24 (-1.05,0.94)	0.45 (-0.34,1.37)	0.009
**Weight-for-length z-score**	56	102		55	102	
< -2 (%)	0 (0.0)	8 (7.8)	0.032	1 (1.8)	4 (3.9)	0.47
≥ -2 (%)	56 (100)	94 (92.2)		54 (98.2)	98 (96.1)	
Median (IQR)	-0.06 (-0.76,1.09)	-0.12 (-0.98,0.54)	0.23	0.31 (-0.58,1.46)	0.36 (-0.52,1.05)	0.89
Median change from baseline (IQR)	-0.18 (-0.69,0.76)	0.23 (-0.29,1.20)	0.031	-0.01 (-0.67,0.95)	0.79 (0.03,1.78)	0.004
**WHO Classification**	56	104		56	104	
Stage 3 or 4 (%)	3 (5.36)	21 (20.2)	0.0122	2 (3.57)	17 (16.3)	0.0172

There was no difference between the groups in the median anthropometric measures of HAZ, WAZ and WLZ at 6 and 12 months (Table 2). However, the median change from baseline in the anthropometric measures was significantly higher in the d4T group relative to the ABC. At 6 months, the d4T group gained significantly more than the ABC group in HAZ (ABC 0.12 vs. d4T 0.34, p = 0.045), WAZ (ABC 0.16 vs. d4T 0.76, p = 0.004) and WLZ (ABC -0.18 vs. d4T 0.23, p = 0.031). Similarly at 12 months, the d4T group had more gain than the ABC in HAZ (ABC -0.24 vs. d4T 0.45, p = 0.009), WAZ (ABC 0.26 vs d4T 1.19, p<0.0001) and WLZ (ABC -0.01 vs. d4T 0.79, p = 0.004).

There was no association between treatment group and virological failure controlling for baseline measures of WAZ; participant age at treatment initiation and the year of treatment initiation. (Table 3).

**Table 3:** Baseline factors associated with virological failure.

Variable	Univariate		Multivariate	
	OR (95% CI)	p-value	OR (95% CI)	p-value
**Study Group**				
ABC	1.5566 (0.8716–2.7798)	0.1348	2.3546 (0.8204–6.7582)	0.1114
D4T	Ref		Ref	
**CD4%**				
CD4% ≤ 25%	0.8106 (0.4698–1.3988)	0.4507		
CD4% > 25%	Ref			
**WHO Stage**				
Stage 3 or 4	1.1055 (0.6130–1.9936)	0.7389		
Stage 1 or 2	Ref			
**Weight-for-age**				
< -2	1.3925 (0.8047–2.4097)	0.2367	1.5833 (0.8884–2.8218)	0.1191
> -2	Ref		Ref	
**Age (months)**	0.9609 (0.9165–1.0073)	0.0974	0.9553 (0.9121–1.0005)	0.0526
**Year of ART initiation**	1.0578 (0.9222–1.2135)	0.4220	0.9075 (0.7153–1.1515)	0.4245

The proportion of children who died was 3.5% in the ABC and 7.9% in the d4T group (p = 0.27). There was no difference in the proportion of children with adherence levels >90% for ABC and d4T in the 2 groups (95% in ABC vs. 86% in d4T, p = 0.10). The proportion of children with adherence >90% for 3TC and LPV/r was similar too (83.6% vs. 87%, p = 0.6957) and (92.73% vs 94%, p = 0.6570) respectively. The log_10_ viral load declined by 0.49 and 0.50 in the ABC and d4T groups respectively, between baseline and the 6 month visit and by 0.27 in both groups over 12 months.

The subgroup analysis of those with baseline viral load over 100 000 copies/ml also showed no difference in the proportion of children virologically suppressed at 6 (ABC 54.5% vs. d4T 68.5%, p = 0.11) and 12 months (ABC 69.8% vs. d4T 74.5%, p = 0.57) (S1 and S2 Tables).

## Discussion

Our analysis showed that overall there was no difference in virological, immunological or clinical outcomes between children receiving ABC or d4T based ART in Soweto, South Africa. The rate of decline of the viral load amongst the groups was also similar.

Even the subgroup analysis of the children with viral load results greater than 100 000 copies/ml showed that both drugs performed equally well. These findings are contrary to what was previously reported in similar settings in South Africa where both a single site and a multi-cohort analysis of programmatic data found worse virological outcomes in ABC based treatment regimens [10, 11]. This may have been due to differences in adherence between the studies. Whereas adherence was not reported in the other studies, both PWP groups had good adherence. There were also differences in ABC access; ABC stock outages were reported by both other cohorts, whereas the children on the PWP were not affected by stock outages.

Our results concur with those found in the CHAPAS-3 study. This RCT compared the NRTIs d4T, AZT and ABC in Ugandan and Zambian children, and found that all three NRTIs performed similarly with regards to clinical, immunological and virological outcomes. In this study, the NRTIs were combined with lamivudine and a NNRTI- either NVP (<3years old) or EFV (>3years old) [12]. Similar findings were also obtained from a systematic review and meta-analysis which concluded that using an ABC containing regimen in ART-naïve children and adolescents was similar in efficacy and safety compared to d4T and AZT [13]. Although the PWP is a programmatic cohort, it is run from within a well-established research unit by health care workers trained in the implementation of clinical trials. This results in the stringent management of patients with regards to implementation of the guidelines, giving attention to missed visits as well as adherence counseling at every visit.

Of note, the lower median log_10_ viral load values seen in the ABC group as compared to the d4T group can possibly be explained by the change in sensitivity of the viral load tests over the years. During the earlier years of the study, the viral load results were reported with a sensitivity of <400 copies/ml. (We therefore used a value of >400copies/ml to define virological failure). With time, the sensitivity of the test was improved and results were reported with a sensitivity of <40copies/ml and even <20copies/ml. As the ABC children were seen in the later time period, a greater number of the patients in this group who had achieved viral suppression, had lower viral load values reported as compared to the patients who had achieved virological suppression in the d4T group and these lower values would contribute to the median log_10_ viral load value being lower.

The notable difference in the baseline characteristics seen in the ABC group, namely the anthropometric measures and clinical staging may be attributed to secular changes as the cohorts belonged to different time periods. A multi-cohort analysis also found better baseline anthropometric characteristics in the ABC group, namely WAZ and HAZ.[11] As the d4T containing regimens were used at an earlier time, these children were diagnosed and initiated on ART-treatment later—after there was a clinical or immunological progression of disease. This would explain the worse clinical staging as well as the lower WAZ and WLZ scores that were seen at baseline in the d4T group. ABC containing regimens were used at a time when there was a scaling up of screening programmes to allow for the early diagnoses and treatment of HIV disease in children. Hence, these children were still clinically well when ART was initiated and it would be expected that their anthropometric measures as well as their clinical staging would reflect this.

The advanced WHO staging in the d4T group persisted at 6 and 12 months post ART-initiation. This may be due to the methodology of WHO staging which continued the baseline stage until resolution of all conditions within that staging category, for example, a child with pulmonary tuberculosis would remain WHO stage 3 from diagnosis until one year after completing treatment.

In contrast, while we found significant differences in the anthropometric measures pre-ART, there was no significant difference in the WAZ; HAZ; and WLZ at both 6 and 12 months on treatment. Children on d4T, who were disadvantaged pre-ART, had remarkable catch-up growth. This is similar to growth responses to ART evaluated in 12 programmes in Malawi, Zambia, Zimbabwe, Mozambique, and South Africa, which showed that lower baseline values for WAZ, HAZ and weight-for-height Z-score correlated with faster catch-up growth on ART [14]. An additional cohort study of HIV-infected children in rural Zambia also showed greater increases in WAZ in the first 6 months for children who were underweight at the time of treatment initiation [15]. Another study in Malawi showed that baseline WAZ and HAZ scores were the most important determinants of growth trajectories on ART [16].

There was no difference in mortality between the d4T and ABC groups. The majority of deaths in the d4T group occurred within the first 6 months of ART. This is in keeping with more advanced disease at the time of ART initiation demonstrated by their WHO stage pre-ART.

The study had several limitations. We included all children who were initiated on ABC that met the inclusion criteria and matched them with children on d4T (see Methods), however, as our numbers were relatively small the power to detect definitive differences may have been limited. Cases were matched in age group bands of a year. Our study had a short duration of follow up which precludes generalizability to long term outcomes. As the groups compared were seen over a different time period, there was also a selection bias due to changes in the pediatric ART guidelines. Prior to the recommendation to initiate all children on ART regardless of CD4 or clinical stage, children needed to have immunological or clinical disease progression in order to qualify for ART. In the PWP, the change in ART initiation criteria occurred around the same time as the change from d4T to ABC based therapy. In other words, d4T was generally started in children with more advanced disease whereas ABC was started in all children.

## Conclusions

ABC and d4T based ART regimens are equally efficacious in young children in Soweto when adherence is good. Children with lower pre-ART anthropometric values experience more remarkable catch-up growth. It is reassuring that, at least in the short term, in this group of patients, the treatment outcomes were similar between those taking ABC compared to d4T-containing regimens.

## Supporting information

S1 Table Outcomes at 6 and 12 months for those with viral loads above 100 000 copies/ml. (RTF)

S2 Table Outcomes at 6 and 12 months for those with viral loads above 100 000 copies/ml. (RTF)

S1 File Supporting data. (XLSX)
