# Supplementary material for: A retrospective case-cohort study comparing treatment outcomes in abacavir versus stavudine containing first line antiretroviral treatment regimens in children <3yrs old, at a paediatric programme based in Soweto, South Africa
Source: PLoS One. 2017 Jul 7;12(7):e0180645. doi: 10.1371/journal.pone.0180645 (PMC5501584; doi:10.1371/journal.pone.0180645)
Supplement: S1 Table — (RTF) [file pone.0180645.s001.rtf]

Table S1. Characteristics at enrolment for those with viral loads above 100 000 copies/ml

Variables	Overall	ABC	d4T	P-Value	
					
Number enrolled	152	46	106		
					
Gender					
Male(%)	68 (44.7)	23 (50.0)	45 (42.5)	0.3900	
Female (%)	84 (55.3)	23 (50.0)	61 (57.5)		
					
Age (years) at HAART start					
0-1 (%)	134 (88.2)	40 (87.0)	94 (88.7)		
1-2 (%)	15 (9.9)	5 (10.9)	10 (9.4)		
2-3 (%)	3 (2.0)	1 (2.2)	2 (1.9)		
Median (IQR) age in months	3.11 (1.98,6.05)	2.55 (1.95,4.86)	3.36 (2.05,6.25)	0.4581	
					
Weight-for-Age z-score					
< -2 (%)	63 (41.4)	14 (30.4)	49 (46.2)	0.0694	
=> -2 (%)	89 (58.6)	32 (69.6)	57 (53.8)		
Median (IQR)	-1.69 (-2.90,-0.61)	-1.20 (-2.10,-0.45)	-1.86 (-3.25,-0.83)	0.0071	
					
Height-for-Age z-score					
< -2 (%)	80 (52.6)	20 (43.5)	60 (56.6)	0.1365	
=> -2 (%)	72 (47.4)	26 (56.5)	46 (43.4)		
Median (IQR)	-2.09 (-2.97,-1.01)	-1.85 (-2.90,-0.89)	-2.25 (-3.29,-1.15)	0.1551	
					
Weight-for-Length z-score					
< -2 (%)	21 (13.9)	4 (8.7)	17 (16.2)	0.2206	
=> -2 (%)	130 (86.1)	42 (91.3)	88 (83.8)		
Median (IQR)	-0.29 (-1.24,0.50)	0.24 (-0.61,0.92)	-0.58 (-1.41,0.21)	0.0016	
					
BMI-for-age z-score					
< -2 (%)	31 (20.4)	5 (10.9)	26 (24.5)	0.0549	
=> -2 (%)	121 (79.6)	41 (89.1)	80 (75.5)		
Median (IQR)	-0.80 (-1.70,0.09)	-0.25 (-0.95,0.38)	-1.19 (-1.87,-0.07)	0.0042	
					
CD4%					
<= 25% (%)	82 (53.9)	25 (54.3)	57 (53.8)	0.9480	
> 25% (%)	70 (46.1)	21 (45.7)	49 (46.2)		
Median (IQR)	23.95 (16.40,34.06)	24.27 (14.07,34.11)	23.95 (17.50,34.00)	0.6795	
					
CD4 Count (cells/µL)					
< 500	20 (13.2)	7 (15.2)	13 (12.3)	0.6207	
=> 500	132 (86.8)	39 (84.8)	93 (87.7)		
Median (IQR) CD4 Count	1290 (878.0,1992)	1485 (807.0,2578)	1266 (878.0,1819)	0.1109	
					
Viral Load (copies/ml)					
< 100,000 (%)					
=> 100,000 (%)					
Log viral load	5.88 (5.74,5.88)	6.03 (5.52,6.42)	5.88 (5.77,5.88)	0.0169	
					
CDC Classification					
Normal (%)	89 (58.9)	34 (73.9)	55 (52.4)		
A (%)	23 (15.2)	7 (15.2)	16 (15.2)		
B (%)	25 (16.6)	4 (8.7)	21 (20.0)		
C (%)	14 (9.3)	1 (2.2)	13 (12.4)		
					
WHO Classification					
Stage 1 or 2 (%)	101 (67.8)	38 (82.6)	63 (61.2)	0.0097	
Stage 3 or 4 (%)	48 (32.2)	8 (17.4)	40 (38.8)		
					
