# Supplementary material for: A retrospective case-cohort study comparing treatment outcomes in abacavir versus stavudine containing first line antiretroviral treatment regimens in children <3yrs old, at a paediatric programme based in Soweto, South Africa
Source: PLoS One. 2017 Jul 7;12(7):e0180645. doi: 10.1371/journal.pone.0180645 (PMC5501584; doi:10.1371/journal.pone.0180645)
Supplement: S2 Table — (RTF) [file pone.0180645.s002.rtf]

Table S2.  Outcomes at 6 and 12 months for those with viral loads above 100 000 copies/ml

Variables	6 Months	12 Months	
	ABC	d4T	P-Value	ABC	d4T	P-Value	
							
Height-for-age z-score	45	95		45	96		
< -2 (%)	16 (35.6)	37 (38.9)	0.6992	22 (48.9)	34 (35.4)	0.1275	
> -2 (%)	29 (64.4)	58 (61.1)		23 (51.1)	62 (64.6)		
Median (IQR)	-1.70 (-2.45,-0.92)	-1.51 (-2.68,-0.80)	0.9964	-1.94 (-2.72,-1.11)	-1.71 (-2.45,-0.73)	0.1752	
							
HAZ Change from baseline	45	95		45	96		
< 1 (%)	31 (68.9)	53 (55.8)	0.1395	20 (44.4)	51 (53.1)	0.3366	
=> 1 (%)	14 (31.1)	42 (44.2)		25 (55.6)	45 (46.9)		
Median change from baseline (IQR)	0.15 (-0.52,0.57)	0.50 (-0.42,1.28)	0.0568	-0.04 (-1.03,1.03)	0.53 (-0.31,1.37)	0.0314	
							
Weight-for-age z-score	45	95		45	96		
< -2 (%)	8 (17.8)	20 (21.1)	0.6510	8 (17.8)	10 (10.4)	0.2221	
> -2 (%)	37 (82.2)	75 (78.9)		37 (82.2)	86 (89.6)		
Median (IQR)	-0.93 (-1.43,-0.01)	-1.17 (-1.87,0.03)	0.3580	-0.79 (-1.43,-0.03)	-0.64 (-1.43,0.22)	0.6235	
							
WAZ Change from baseline	45	95		45	96		
< 1 (%)	27 (60.0)	48 (50.5)	0.2939	26 (57.8)	38 (39.6)	0.0431	
=> 1 (%)	18 (40.0)	47 (49.5)		19 (42.2)	58 (60.4)		
Median change from baseline (IQR)	0.38 (-0.29,1.41)	0.83 (-0.03,1.70)	0.0444	0.45 (-0.29,1.45)	1.26 (0.40,2.13)	0.0012	
							
Weight-for-height z-score	45	94		44	94		
< -2 (%)	0 (0.0)	7 (7.4)	0.0603	1 (2.3)	4 (4.3)	0.5614	
> -2 (%)	45 (100)	87 (92.6)		43 (97.7)	90 (95.7)		
Median (IQR)	-0.08 (-0.78,1.46)	-0.12 (-0.93,0.50)	0.4075	0.31 (-0.60,1.02)	0.36 (-0.46,1.02)	0.8640	
							
WLZ Change from baseline	45	93		44	93		
< 1 (%)	27 (60.0)	52 (55.9)	0.6492	25 (56.8)	39 (41.9)	0.1030	
=> 1 (%)	18 (40.0)	41 (44.1)		19 (43.2)	54 (58.1)		
Median change from baseline (IQR)	-0.10 (-0.64,0.92)	0.23 (-0.25,1.42)	0.1166	-0.05 (-0.80,1.21)	0.84 (0.03,1.78)	0.0128	
							
BMI z-score	45	95		45	96		
< -2 (%)	0 (0.0)	7 (7.4)	0.0617	0 (0.0)	3 (3.1)	0.2307	
> -2 (%)	45 (100)	88 (92.6)		45 (100)	93 (96.9)		
Median (IQR)	-0.05 (-0.82,1.43)	0.07 (-0.77,0.73)	0.6346	0.50 (-0.57,1.42)	0.63 (-0.10,1.38)	0.7268	
							
BMIZ Change from baseline	45	95		45	96		
< 1 (%)	25 (55.6)	44 (46.3)	0.3071	20 (44.4)	33 (34.4)	0.2498	
=> 1 (%)	20 (44.4)	51 (53.7)		25 (55.6)	63 (65.6)		
Median change from baseline (IQR)	0.49 (-0.24,1.52)	0.78 (0.15,1.95)	0.1452	1.12 (-0.13,1.72)	1.48 (0.65,2.55)	0.0229	
							
CD4%	42	93		42	93		
<=25% (%)	13 (31.0)	24 (25.8)	0.5349	9 (21.4)	14 (15.1)	0.3617	
>25% (%)	29 (69.0)	69 (74.2)		33 (78.6)	79 (84.9)		
Median (IQR)	29.65 (23.42,38.90)	31.00 (25.00,35.62)	0.9659	31.70 (26.39,38.90)	32.19 (28.00,37.66)	0.9394	
							
CD4 Count (cells/µL)	43	93		42	93		
< 500 (%)	0 (0.0)	1 (1.1)	0.4949	0 (0.0)	2 (2.2)	0.3383	
=> 500 (%)	43 (100)	92 (98.9)		42 (100)	91 (97.8)		
Median (IQR)	1943.0 (1449.0,2971.0)	1824.0 (1365.0,2483.0)	0.2318	1922.0 (1291.0,3004.0)	1854.0 (1378.0,2451.0)	0.4513	
							
Viral Load	44	92		43	94		
< 400 (%)	24 (54.5)	63 (68.5)	0.1133	30 (69.8)	70 (74.5)	0.5652	
=> 400 (%)	20 (45.5)	29 (31.5)		13 (30.2)	24 (25.5)		
Median Log10 VL (IQR)	2.44 (1.60,3.08)	2.60 (1.85,2.90)	0.3032	2.24 (1.60,3.45)	2.60 (1.70,2.62)	0.2009	
							
